# How Can the Current State of AI Guide Future Conversations of General Intelligence?

**DOI:** 10.3390/jintelligence12030036

**Published:** 2024-03-20

**Authors:** Tomoe Kanaya, Ali Magine

**Affiliations:** 1 Department of Psychological Science, Claremont McKenna College, Claremont, CA 91711, USA; 2 Independent Researcher, Raleigh, NC 27695, USA; alimagine@fundamentali.com

**Keywords:** artificial intelligence, human intelligence, general intelligence

## Abstract

Similar to the field of human intelligence, artificial intelligence (AI) has experienced a long history of advances and controversies regarding its definition, assessment, and application. Starting over 70 years ago, AI set out to achieve a single, general-purpose technology that could overcome many tasks in a similar fashion to humans. However, until recently, implementations were based on narrowly defined tasks, making the systems inapplicable to even slight variations of the same task. With recent advances towards more generality, the contemplation of artificial general intelligence (AGI) akin to human general intelligence (HGI) can no longer be easily dismissed. We follow this line of inquiry and outline some of the key questions and conceptual challenges that must be addressed in order to integrate AGI and HGI and to enable future progress towards a unified field of general intelligence.

## 1. What Have AI People Been Doing? Machine Learning (ML) and Large Language Models (LLMs)

The main goal of artificial intelligence (AI) research is to engineer more capable AI systems, ultimately creating a single system that can perform any task that requires intelligence at the same level as or exceeding the proficiency level of a human. The majority of the AI advances in the past two decades have stemmed from a particular subfield of AI, known as machine learning (ML), which is any system where measurable performance can be improved upon just by consuming more data (Mitchell 1997). In order to train an ML system, a developer must have a mathematically specified objective for the system. During the training, an ML system receives a score based on that objective function and tries to compute the best internal changes it can make in order to obtain a higher score later (Russell and Norvig 2020). 

For instance, if the objective of the system is to achieve a higher score in a Go match, one can assign “winning at Go” as an apparent goal for the system. Through the training stage, the system would receive a score based on each Go performance, ultimately learning to achieve higher scores by improving its performance in the game of Go. Such a system cannot play a match of Chess, a game that (arguably) requires less-demanding cognitive skills, no matter how well it performed at Go (Silver et al. 2016). For that reason, all AI systems we have been able to implement so far can be considered ‘narrow’ AI. 

In contrast, AI researchers seek to build a single system that can perform many things without any need to specify, or to explicitly train for, the task in advance. This ability in such a system has been coined artificial general intelligence (AGI; Goertzel and Pennachin 2006), as an analogy to human general intelligence (HGI). For almost all AI researchers, AGI remained elusive until quite recently, when powerful Large Language Models (LLMs), such as GPT4, were publicly released. 

Current LLMs are trained by next-token prediction with giant models on very large amounts of data. Similar to previous, smaller ML models, LLMs are also trained by a single objective for the system (Zhao et al. 2023). That reason could be used to still justify considering LLMs to be narrow AI. Yet, the model has gained the ability to perform an unbounded number of ML tasks (although not an unbounded number of human tasks), whereas previous models needed explicit training for a single or just a few tasks. Optimizing one single, but highly abstract, objective seems to achieve intelligent behavior that was not expected previously, which suggests that our reasoning to judge the generality of AI systems needs to be revisited.

These broad capabilities of powerful LLMs have reignited debates over how soon AGI will be achieved, or whether the state-of-the-art LLMs possess any general intelligence at all. The debates cover various aspects of intelligence. For example, Franceschelli and Musolesi (2023) argue that LLMs can create content that meets the highly cited creativity criteria developed by Boden (1991), which require that created products are “new, surprising and valuable.” They admit, however, to only observe weak forms of creativity as opposed to transformational creativity, likely due to a lack of processes such as motivation. Andreas (2022) argues that LLMs possess intentions but in the narrow sense as modules within communicative agents. There are also some claims that AGI has already been achieved (Wei et al. 2022) and that these models have shown actual signs of intelligence (e.g., Bubeck et al. 2023) by citing selected cases of impressive performance on previously challenging ML tasks. Others argue that, while the recent advances are very impressive, it is still premature to conclude that AGI has been achieved or that it will be achieved soon (Mitchell 2023). For example, Mitchell and Krakauer (2023) argue that LLMs lack true understanding by showing documented mistakes that humans would likely never make.

Indeed, there is no unanimous agreement in the AI field on any working definition of intelligence, which has prevented the field from converging on any accepted benchmark as a valid test for general intelligence. The concept of general intelligence itself remains undefined in AI with only one available example, the general intelligence in humans (e.g., Hernández-Orallo 2017b; Magine 2022; Moskvichev et al. 2023). The increased interest in AI among the public has pressured governments to create proper regulations and has hastened the need to clarify and understand the differences and similarities between AGI and HGI. These clarifications are needed to be able to formulate existing debates in better ways, allowing further progress on current and future debates.

## 2. Challenges in Comparing AI and HI

Making direct comparisons between the advances in AGI and HGI remains challenging because of the different ways both constructs have evolved and been assessed throughout their respective histories. While it was easier to ignore these differences in the past due to AI’s lack of impressive capabilities, this is clearly no longer the case. Below are (some of) the challenges that are currently preventing AGI and HGI from getting on an equal footing and thereby informing each other in an effective manner. 

### 2.1. Benchmarks and Reporting on Intelligence

Just like any other field of scientific research, progress needs to be measured using benchmarks and defined outcomes. However, since the performance of previous AI systems was poor, the reporting of results in AI engineering focused on what an AI did right as opposed to what it did not. For instance, a report that cites a very high accuracy of an image classification system on a large dataset consisting of many classes may fail to report particular instances of misclassification (such as higher misclassifications for female faces compared to male faces), that can contain more insights about the true competency of the AI system (Burnell et al. 2023). This approach makes it difficult to develop a true understanding of the level of intelligence and limitations of an AI system. With recent advances in AI, the need to rethink the reporting of the intelligence of these systems closer to or on par with human intelligence science has become more important than before (Burnell et al. 2023). 

HGI, on the other hand, has over a century of research with IQ tests and other intelligence assessments that allow for standardized benchmarks and outcomes. While there are significant controversies surrounding the use and interpretation of these measures, especially within the context of culturally diverse and under-represented populations (e.g., Jonson and Geisinger 2022), these measures provide scores based on a norming distribution that can have immediate ‘real-world’ interpretations and implications for humans. For example, an individual’s IQ score plays a prominent role in their disability status and educational opportunities, including Intellectual Disability and Learning Disability diagnoses and entrance into Gifted programs (Kanaya et al. 2022). 

Creating formal ways to connect AGI and HGI benchmarks and measurements will enable opportunities to expand upon our knowledge of both intelligences. Notable collaborations have already been made with neuroscience, allowing for substantial advances in our understanding of human neural activity (Macpherson et al. 2021). For example, artificial neural networks have been used to understand and measure neural activity related to the brain’s visual system (e.g., Federer et al. 2020; Jacobs and Bates 2019). Similar advances could lead to a better understanding of the age-related differences in human memory and vocabulary performances (e.g., Baltes et al. 2006) or sex differences in the tails of the HGI distributions (Halpern and Kanaya 2020). In addition, extending benchmarks for multi-modal LLMs (LLM models that can take and generate text, image, music, etc.) could assist in developing standardized assessments for human abilities that have been difficult to measure in the past, such as assessments of neuro-atypical humans (e.g., Banire et al. 2023).

### 2.2. Factoring in the Effect of Methodology

Even if benchmarks are established and achieved, an AI system can perform a task via methods that humans would consider shortcuts. Having vastly more storage, computing power, and speed is often the enabler, allowing for methodologies that humans may not consider true intelligence. For instance, for games such as Chess or Go, having superhuman ability to store and crunch a vast number of branches in the game tree of possibilities within seconds makes the stellar performance of the AI system unrelatable to human intelligence (Hernández-Orallo 2017a). While there have been multiple attempts to develop assessments that can serve as IQ test equivalents for machines (e.g., allowing people to determine which AI system is ‘smarter’ than the other), these efforts have not seen massive success (e.g., Chollet 2019; Moskvichev et al. 2023). 

If two systems perform similarly across an array of tasks, the internal efficiency with which the performance has been achieved should be considered when determining which system is more intelligent. Humans have access to vastly smaller resources (e.g., short-term memory, computational power, exposure to training data, etc.) compared to machines, and therefore, they tend to utilize more efficient methodologies than current AI systems. But, the way to account for differences in the efficiency of a methodology is not clear and must be identified in order to make comparisons between AGI and HGI.

### 2.3. Factoring in the Priors

A prior can be defined as any *internal* preparedness that a cognitive system has a priori for performing any task (Griffiths et al. 2008). For example, brain architecture, initial wiring in humans, and their particular sensory apparatus are rich sources of priors. Core knowledge, which enables humans to understand and learn about objects, language, spatial navigation, and numeracy from birth, could also be seen as priors that have a direct relationship with HGI (Spelke and Kinzler 2007). 

In AI, the architecture of a computation model or hand-designed elements in the training process are sources of encoding priors. Powerful and useful priors can be encoded into machines to fool humans of general competency, yet can lack the generality that HGI may possess. For example, humans know the position of objects does not affect the identity of objects. This very useful fact can be inserted into the architecture of an AI model by what is known as convolutional filters. They are used in the most well-known AI vision systems, namely, Convolutional Neural Networks (CNNs), which are widely used in the self-driving car technology stack. While a CNN leverages translational symmetry in the world via encoding this prior, perhaps it still misses other priors compared to humans’ much more robust visual system (Anselmi and Poggio 2022).

The vision system of self-driving cars can be brittle and prone to being fooled in non-human ways (Zhang et al. 2023). To compensate, these systems need vastly more training data (many millions of miles of driving data) than a human would need to be reliable (Chen et al. 2023). Therefore, it is important to study the priors that humans and AI systems use further and to take them into account when comparing different AI systems or making conclusions between AGI and HGI.

### 2.4. Environmental Influences

While our priors may prepare us well for certain tasks, the environment can interact with us in a variety of ways, boosting our ability to leverage our priors in some situations and rendering them useless if the environment changes too much in other situations. For example, some AI systems may have access to internet-scale data created by human users around the world, while others may only have access to proprietary data purchased by the developers of the system, and others may have restricted access to that proprietary data (e.g., for copyright reasons). A fair comparison of these two types of systems in their performance on tasks, no matter how generic, may not be a straightforward task. The element of receiving external help, developmentally or otherwise, remains among the most ignored facets in the broader discussion of machine intelligence. 

The field of HGI has plenty of experience in this debate. Indeed, environmental influences have provided valuable insights into understanding HGI. For example, in the Carolina Abecedarian Early Intervention Project, low-income children in North Carolina were provided intensive, high-quality, educational experiences for 5 years. Longitudinal analyses revealed that the positive impact of this environment resulted in significant improvements, including increases in HGI, neurological activity, and adulthood outcomes (Nisbett 2009). 

The research literature on stereotype threat has shown that smaller changes in the environment can also lead to significant changes in HGI. Humans underperform on ability tests when they believe they may be confirming a negative stereotype about their identity, such as Black Americans and women on math tests or older people on a memory test. Simple interventions, such as de-emphasizing a person’s identity status (e.g., not asking for a person’s race or gender), can reduce the threat and improve cognitive performance (Spencer et al. 2016). 

In addition, the Flynn effect refers to the global rise in IQ seen over the last 70 years (Flynn 2012). The size of these gains varies based on the type of assessment. Specifically, gains are higher in tests that measure fluid intelligence, such as the Ravens Matrices, but are very small on tests of crystallized intelligence, such as the Information subtest of the Wechsler IQ norms (Kanaya and Ceci 2011). Recently, the Flynn effect has shown plateaus and reversals in some countries (Dutton et al. 2016; Vainikainen and Hautamäki 2022), emphasizing the dynamic role of the environment in HGI and the need to develop a better understanding of the various roles of the environment in interpreting AGI and HGI performance (Flynn 2012).

While the Flynn effect gains have been slow and systematic, AI gains have increased rapidly over the past few decades, and future advances could serve as a source of stronger Flynn effect gains. Further, the reasons behind the advances in AI have been extensively documented, while the reasons behind the Flynn effect are still poorly understood (e.g., Trahan et al. 2014). Charting the longitudinal advances within AI with the longitudinal trends within the Flynn effect by location, task, and cohort (e.g., Kanaya et al. 2005; O’Keefe and Rodgers 2020) could provide valuable insights into the trajectory of HGI across the lifespan and the ways in which human gains can lead to technological gains and vice versa.

### 2.5. Human vs. Artificial Errors

Current, state-of-the-art AI systems are based on autoregressive LLMs, which are models that complete sequences one token at a time. Each token is conditioned on the previous set of tokens including the last-generated one (Zhao et al. 2023). There is no planning and backtracking involved in auto-regression, which is a big limitation. These models are designed and trained to maximize the plausibility of the generated information, not its truthfulness. Currently, AI researchers do not have a good mechanism to make these models prioritize truth over plausibility, leading to errors that are easily detected by humans, commonly referred to as hallucinations (Rawte et al. 2023). In other words, as the model learns more, it can understand less (e.g., Azaria and Mitchell 2023; Mitchell 2023). For this reason, current LLM systems are very sensitive to how they are prompted in ways that humans are not. More specifically, two different phrasings of the same prompt can result in vastly different responses from the AI, while being perceived similarly by humans (Sclar et al. 2023).

On the other hand, humans are sensitive to variations in content references during decision making in ways that AIs can easily avoid. For example, research on priming and interference has shown that exposure to a stimulus can influence human memory retrieval and lead to decision-making errors, especially if decisions are made quickly (Kahneman 2011). Algorithms, however, can easily avoid these errors without requiring the slower processing time that humans usually need to avoid these errors. In other words, the types of mistakes that can be expected from ‘smart humans’ versus ‘smart machines’ are not the same. Acknowledging and identifying the specific parameters of this truth and error gap will be important for future AGI-HGI comparisons. 

### 2.6. Goals and Agency 

Humans have opportunities for choosing their goals and experience benefits from these choices. Educational research has consistently shown that parental and student agency can lead to significant improvements in educational attainment and other learning outcomes (e.g., Wong et al. 2023). Dickens and Flynn (2001) have also proposed a social-multiplier model, where small changes, such as choosing an activity that provides strong environmental support for HGI, can multiply and lead to large and systemic HGI gains.

ML systems, however, are trained for a single overall objective. While this task can be highly abstract, leading the system to some general abilities, machines still lack the ability to choose their own overall goal or to decide what would be beneficial to pursue (Summerfield 2022). From intelligence measurement and comparison to HGI perspectives, it is unclear how to factor this fundamental ability, or lack thereof, into intelligence. While it is arguable that we may not need machines that can choose their own goals, understanding the effects of having a choice within HGI will be important and inspirational for understanding how we can and should incorporate such a capability into AI systems and for advancing their intelligence.

### 2.7. Accounting for Vastly Different Scopes

One can consider a calculator a very narrow AI system. However, carrying out fast arithmetic on very large numbers is not the *scope* of HGI, which many researchers (e.g., Nisbett et al. 2012) define as “the ability to reason, plan, solve problems, think abstractly, comprehend complex ideas, learn quickly and learn from experience, to “catch on,” “make sense” of things, or “figure out” what to do”(Gottfredson 1997, p. 13). Similarly, some of the problem-solving abilities observed in biological organisms at the cellular or multicellular levels go beyond what we would ever demand of human intelligence (Levin 2019). For instance, slime molds show signs that they are able to solve complex spatial problems in intelligent ways (Jabr 2012). We may dismiss these examples of intelligence as non-relatable to HGI. However, it is harder to dismiss other tasks that are superhuman extensions of human abilities, such as being able to translate any text in any of the several hundred human languages within seconds or to answer graduate-level questions in hundreds of disciplines. The question of whether or not a larger scope of abilities should be counted towards intelligence is a growing challenge, given the growth in AI advancements. We call this the *scope challenge*, and it has been revived by recent advances in LLMs.

## 3. Discussion and Future Directions

The recent advances in AI have made new opportunities for cross-pollination and comparisons between AGI and HGI that were not possible until now. In order to create an effective and productive dialogue between AGI and HGI, however, a broader understanding of how to measure AGI, with respect to what we know about HGI, is required. Successful integration between AGI and HGI can play an influential role in many advances within general intelligence, including the ability to analyze the unappreciated aspects of an intelligent agent, to develop ways of improving human intelligence, and to develop methods to work around the limitations of human intelligence. In this paper, we have outlined some of the challenges and obstacles to this broader understanding. While this is not an exhaustive list, we hope it will serve as a useful primer for identifying challenges that need to be addressed before we can meaningfully combine AGI and HGI. Future research within AGI *and* HGI should focus on embracing these challenges and allowing them to expand our lines of inquiry in gaining a deeper understanding of intelligence and its underpinnings.
